# Hydrogen Production via Steam Reforming: A Critical Analysis of MR and RMM Technologies

**DOI:** 10.3390/membranes10010010

**Published:** 2020-01-03

**Authors:** Giovanni Franchi, Mauro Capocelli, Marcello De Falco, Vincenzo Piemonte, Diego Barba

**Affiliations:** 1 Unit of Process Engineering, Department of Engineering, Università Campus Bio-Medico di Roma, via Álvaro del Portillo 21, 00128 Rome, Italy; m.capocelli@unicampus.it (M.C.); m.defalco@unicampus.it (M.D.F.);; 2 Unit of Chemical-physics Fundamentals in Chemical Engineering, Department of Engineering, Università Campus Bio-Medico di Roma, via Álvaro del Portillo 21, 00128 Rome, Italy; v.piemonte@unicampus.it

**Keywords:** membrane, methane, hydrogen permeation, mathematical model, conversion

## Abstract

‘Hydrogen as the energy carrier of the future’ has been a topic discussed for decades and is today the subject of a new revival, especially driven by the investments in renewable electricity and the technological efforts done by high-developed industrial powers, such as Northern Europe and Japan. Although hydrogen production from renewable resources is still limited to small scale, local solutions, and R&D projects; steam reforming (SR) of natural gas at industrial scale is the cheapest and most used technology and generates around 8 kg CO_2_ per kg H_2_. This paper is focused on the process optimization and decarbonization of H_2_ production from fossil fuels to promote more efficient approaches based on membrane separation. In this work, two emerging configurations have been compared from the numerical point of view: the membrane reactor (MR) and the reformer and membrane module (RMM), proposed and tested by this research group. The rate of hydrogen production by SR has been calculated according to other literature works, a one-dimensional model has been developed for mass, heat, and momentum balances. For the membrane modules, the rate of hydrogen permeation has been estimated according to mass transfer correlation previously reported by this research group and based on previous experimental tests carried on in the first RMM Pilot Plant. The methane conversion, carbon dioxide yield, temperature, and pressure profile are compared for each configuration: SR, MR, and RMM. By decoupling the reaction and separation section, such as in the RMM, the overall methane conversion can be increased of about 30% improving the efficiency of the system.

## 1. Introduction

Hydrogen is widely used in industrial sector such as oil refining, ammonia and methanol synthesis, iron and steel production [1], and its production has fourfold from 1975 to 2018, reaching 115 Mton/y. Nowadays, over 95% of hydrogen is obtained from fossil fuels, consequently releasing about 830 million tons of carbon dioxide per year [2]. 

The 48% of current hydrogen production is via steam reforming of natural gas (SR), 30% via petroleum fraction, 18% via coal gasification, and only 4% via electrolysis due to the still high cost of production (2.50–5.30 US$/gge against 1.33–2.30 US$/gge of SR) [3,4,5,6]. Moreover, the production through electrolysis requires the use of electricity, still and for many years to come, heavily linked to the combustion of fossil fuels. Other processes able to produce hydrogen from renewable resources such as aqueous phase reforming, photoelectrolysis and thermochemical water splitting are at laboratory scale [7,8,9,10]. Biomass gasification has been developed at commercial scale, but hydrogen production is still expensive compared to SR (reaching in some cases 3.5 US$/gge) [11,12,13].

To meet improved targets of costs and efficiency of the decarbonization pathways, this group has contributed to find an attractive process scheme for high-performance and CO_2_-free hydrogen production. The results from this R&D group, mainly in collaboration with ENEA and KT-Technologies, focused on two main research lines:

(i) separation technology to produce both a high purity hydrogen and a high-pressure CO_2_ stream at the basis of the pre-combustion capture schemes [14,15];
(ii) innovative process scheme to couple the heat demanding thermochemical conversion with renewable energy [16,17,18].

As a recent example, CoMETHy (Compact Multifuel-Energy to Hydrogen converter) Project, co-funded by the European Commission under the Fuel Cells and Hydrogen Joint Undertaking (FCH JU), has showed the feasibility of a process scheme, powered by concentrating solar power (CSP) plants using molten salts as heat transfer fluid, to produce pure hydrogen (chemical storage of solar energy) [17,19]. 

The main innovative process scheme, also adopted by this research group in the cited R&D projects, includes hydrogen separation through palladium membranes to increase the reaction yield [20,21] in two different configurations: the membrane reactor (MR) and the reformer and membrane module (RMM). Both schemes can overcome the thermodynamic limit of SR by removing the produced hydrogen and enabling strategies towards CO_2_ capture [15,16,20,21,22,23,24]. 

In the MR configuration, the hydrogen is continuously collected from the reaction environment to the permeate side of a membrane put inside the reactor. In the simplest configuration, the catalyst is packed in the annular zone while the membrane represents an inner concentering tube. A sweeping gas may be fed through the inner tube, co-currently or counter-currently, in order to increase the driving force and to promote the hydrogen separation. 

The reformer and membrane module (RMM) configuration presents the alternation of multiple reaction stages with multiple separation stages: an hydrogen selective membrane is interposed between two reaction units. By decoupling reaction and separation, it is possible to optimize the heat transfer in the reaction zone and the mass transfer in the separation zone separately. As an example, to adopt milder operating conditions for the membranes can increase membrane durability and enable the use of thinner membranes, achieving higher hydrogen separation efficiency. Although less compact than MR, since it requires two devices to carry out reaction and separation, RMM appears more feasible from an engineering point of view. The open architecture of RMM allows to obtain easier maintenance for both the membrane modules and catalyst replacement, which makes this configuration more suitable for industrial scale applications [16,25,26,27]. 

For both configurations, most scientific works are realized at laboratory scales with very few exceptions, as the pilot plant implementing RMM configuration previously studied by this research group [15,25,26,27,28]. 

Several mathematical models have been developed to describe the reaction-permeation phenomena in different geometries at different operating conditions [27,28,29,30,31,32,33,34,35,36,37]. Ward et al. [30] analyzed, for the first time, all the elementary steps regarding hydrogen permeation through Pd self-supported membrane: diffusion in the gas phase, adsorption on the surface, dissociation in atomic hydrogen, diffusion within the metal, recombination of the diatomic molecule and, finally, the desorption from the membrane. Caravella et al. [31,32], studied Pd-alloy membranes, introducing an ‘adjusted’ pressure exponent in the Sievert law, accounting for different operating conditions, non-ideal behaviors and limiting permeation step. Barbieri et al. [33] considered the effect of CO in the gas mixture by means of a Sieverts-Langmuir law, where the reduction of hydrogen permeation through the membrane was a function of the surface coverage by the inert gas. Also, Abir et al. [34] estimated the effect of adsorbents on the membrane considering Langmuir isotherms. Gallucci et al. [35] compared fluidized bed reactors and packed one showing a reduction of gradients concentration and thermal stability with a consequently low membrane surface of about 20–25%. However, due to fluidization, erosion issues and membrane sealing could affect the mechanical stability of the system. Murmura et al. [36] proposed an elegant sensitivity analysis carried out through dimensionless number, addressed the effect of radial gradient in membrane reactors and shown the existence of boundary layer near the membrane surface, whose dimension depend on fluid dynamics conditions. Whereas, Marin et al. [37] implemented a 2D model for mass, heat and momentum balances in case of high hydrogen flux through the membrane.

In our previous work [27], we applied a physical-mathematical model of the mass transfer, in three different geometries, considering both the concentration polarization and the membrane permeation, to simulate the experimental results of more than 70 tests obtained at the Chieti Pilot Plant [25,26]. 

In this work, we use the know-how acquired through the aforementioned experimental and modeling works to propose a kinetic comparison between the three configurations indicated: SR, MR, and RMM. According to the defined mass transfer correlation, the hydrogen permeation, through the membrane and the hydrogen production due to reaction, are compared. Since the two velocity are equal only at the end of the reactor, radial gradients are neglected and one-dimension model for mass, heat, and momentum balances has been proposed for both SR and MR. The commercial steam reformer is simulated according to Xu and Froment data [38,39], but several feedstocks can be considered. In order to make a comparison between the two configurations the same void fraction of the bed, the same geometrical characteristics and the same operating conditions have been considered. The membrane permeability has been estimated taking into account the activation energy for the diffusion of hydrogen atoms, the standard enthalpy and the entropy change due to the dissociation reaction [27]. Only for RMM, where the membrane is outside of the reformer, the separation module is modeled as an isothermal and isobar material exchanger [27]. In Section 3.1, Section 3.2 and Section 3.3 at fixed feed composition, GHSV, S/C, HRF, and membrane area, a comparison between SR, MR and RMM is performed (‘base case’). In Section 3.4, instead, the effect of the main parameters (GHSV, S/C, and HRF) affecting the efficiency of the system in the three configurations is analyzed. 

## 2. Mathematical Modeling

**Figure 1:**
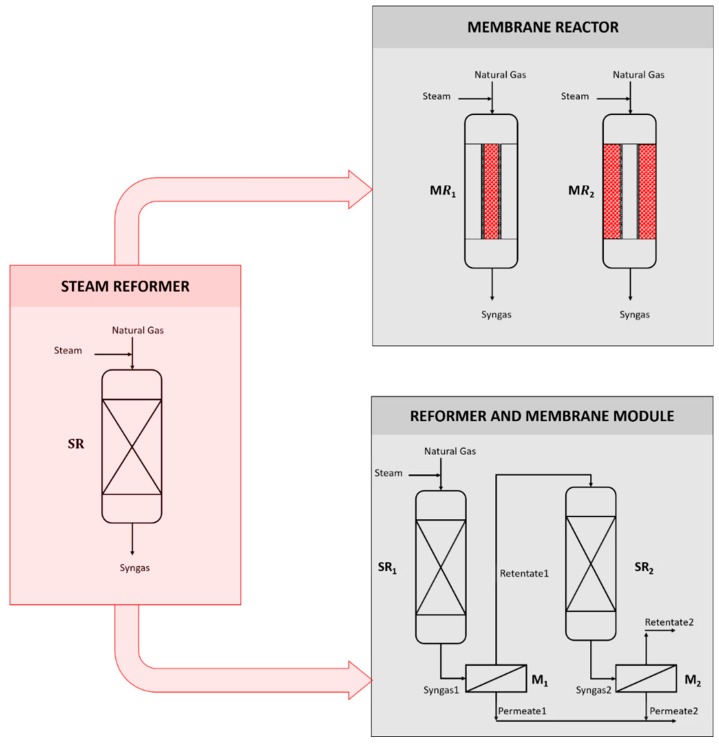
Schematic representation of steam reformer (SR), membrane reactor (MR), and reformer and membrane module (RMM).

The present section describes the differential equations used to model the steam reformer (SR), the membrane reactor (MR), and the reformer and membrane module (RMM), depicted in Figure 1.

The SR is simulated as fixed bed reactor neglecting radial temperature gradients, radial and intraparticle concentration [40]. These hypotheses allow to develop a one-dimensional model on the axial direction for mass, heat, and momentum balances. The overall heat transfer coefficient and friction factor are worked out according to the following expressions [38,41]

(1) $$U={\left[\frac{1}{h_{i}}+\frac{d_{i}}{2k_{t}}\ln\left(\frac{d_{e}}{d_{i}}\right)\right]}^{-1}$$

(2) $$f=\frac{1-\varepsilon }{\varepsilon^{3}}\left[1.75+150\cdot \left(\frac{1-\varepsilon }{Re}\right)\right]$$

where the void fraction of the bed is estimated by means of Haughey and Beveridge correlations [42]. The kinetics, instead, are described by means of Xu and Froment equations, where steam reformer, water gas shift, and the overall steam reformer reactions are considered respectively [39]. The rate of reactions, the kinetic parameters, the equilibrium and adsorption constants are listed in Table 1.

For the MR, considered for this study in a double-pipe configuration enabling the contemporary heat and material exchange, two configurations are possible: one in which the catalyst is packed inside the inner tube (MR1) and, the other one, where the catalyst is in the annulus section (MR2) [16]. In order to develop a proper mathematical model, the rate of hydrogen production and the rate of hydrogen permeation through the membrane, are compared as

(3) $${\left(\frac{dF_{H_{2}}}{dz}\right)}_{prod.}=r_{H_{2}}\eta_{H_{2}}\rho_{c}\Omega$$

(4) $${\left(\frac{dF_{H_{2}}}{dz}\right)}_{perm.}=F_{OG}\left(p_{H_{2}}^{R}-p_{H_{2}}^{P}\right)\pi OD_{t}$$

where the overall mass transfer coefficient contains the contribution of mass transfer coefficient on the retentate side $F_{G}^{R}$ and the membrane permeability $P_{H_{2}}$, neglecting the presence of sweeping gas.

The overall mass transfer coefficient and membrane permeability are calculated according to the expressions

(5) $$F_{OG}={\left[\frac{{\left(P-p_{H_{2}}\right)}_{ML}}{F_{G}^{R}}+\frac{\delta }{P_{H_{2}}}\left(\sqrt{p_{H_{2}}^{LI}}+\sqrt{p_{H_{2}}^{RI}}\right)\right]}^{-1}$$

(6) $$P_{H_{2}}=\frac{1}{2}D_{0,H}\exp\left(\frac{\Delta S_{R}^{0}}{R}\right)\exp\left(-\frac{E_{D}+\Delta H_{R}^{0}}{RT}\right)=P_{H_{2}}^{0}\exp\left(-\frac{E_{a}}{RT}\right)$$

where the activation energy for the diffusion of hydrogen atoms $E_{D}$, the standard enthalpy of the surface dissociation reaction $\Delta H_{R}^{0}$, and the entropy change of the dissociation reaction $\Delta S_{R}^{0}$ are considered [27].

The RMM scheme, as shown in Figure 1, consists of two reactors and separation modules. The reaction section is simulated according to the previous considerations for SR, whereas the membranes are considered as an isothermal and isobar material exchanger.

**Figure 2:**
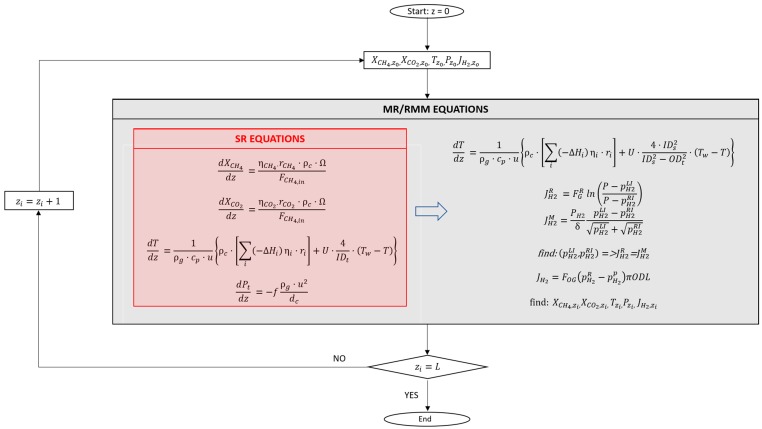
Flow chart of simulation.

The mathematical models are developed in MATLAB for both steam reformers and membrane modules. The domain is divided into 400 elements and the methane conversion, carbon dioxide yield, temperature and pressure profile have been estimated by means of the explicit Runge–Kutta of fourth order method and the Levenberg–Marquardt algorithm respectively (Figure 2). 

In the following section, the numerical results obtained for the three configurations and the related pros and cons of the three options are discussed for a fixed gas hourly space velocity (GHSV), steam to carbon ratio (S/C), and hydrogen recovery factor (HRF). While in Section 3.4 the feed composition is fixed, and the effect of aforementioned parameters is analyzed. 

## 3. Results and Discussion

A commercial steam reformer tube has been simulated according to Xu and Froment data and the main geometrical characteristics and operating conditions are summarized in the Table 2. In order to make a comparison between the three configurations, the same operating conditions, geometrical length, and void fraction of the bed are considered.

The feed, a mixture of CH_4_ (21.28 mol %), CO_2_ (1.19 mol %), H_2_ (2.60 mol %), H_2_O (71.45 mol %), and N_2_ (3.49 mol %), enters the SR, MR, and RMM. The S/C is equal to 3.5. Whereas the GHSV is the same for SR, MR1, and RMM and equal to 11,600 h^−1^. However, for MR2 is about 2660 h^−1^. In this type of reactor, indeed, the catalyst is stacked in the annulus section. Therefore, assuming the same void fraction of the bed and the same geometrical length, the catalyst volume increases. In order to have the same GHSV the shell diameter should be reduced from 0.25 m (of the present case) to 0.16 m with an annulus section of 3 cm. Hence, the simulation has been performed at lower GHSV parameter. 

### 3.1. SR and MR Comparison

**Figure 3:**
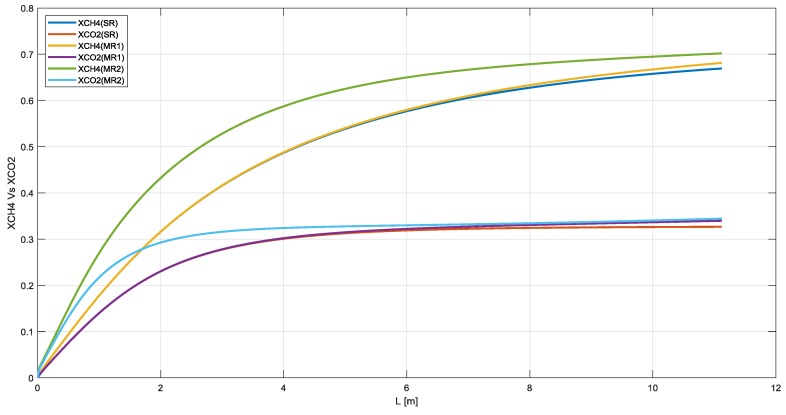
Methane conversion and carbon dioxide yield in SR, MR1, and MR2.

In this section the SR and MR have been analyzed. It is worth to highlight that the methane conversion reaches about 67%, 68%, and 70% for SR, MR1 and MR2 with a carbon dioxide yield of 33%, 34%, and 35% respectively (Figure 3). 

**Figure 4:**
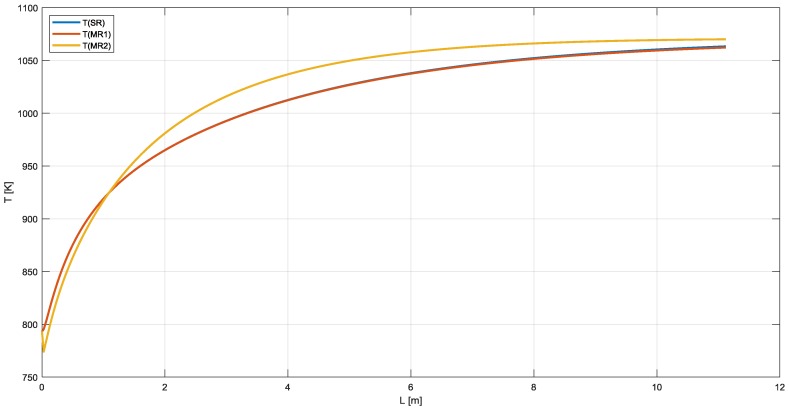
Temperature profile in SR, MR1, and MR2.

As shown in Figure 4, the temperature profile is almost the same for SR, MR1, and MR2 with an average temperature for MR2 higher than the other configurations. The temperature drops at the inlet of the reactor is ordinary in packed bed reactor and can affect the membrane stability. Therefore, a pre-reformer is recommended [35].

**Figure 5:**
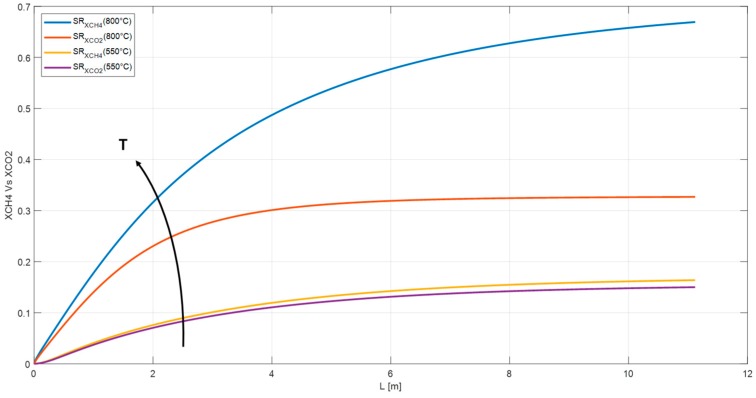
Methane conversion and carbon dioxide yield in a SR operating at 800 °C and 550 °C respectively.

The simulation has worked out at temperature higher than the maximum allowable for a membrane reactor in order to make a comparison between the SR and MR in homogenous conditions. A temperature drops in a SR from 800 °C to 550 °C, indeed, would reduce the methane conversion from 67% to 16% and the hydrogen partial pressure, at the outlet of the reactor, of about one-third from 11.36 bar to 4.32 bar. Lower temperature promotes the water gas shift reaction reducing the efficiency of the system. Figure 5 shows the cited temperature effect on XCH_4_ and XCO_2_. 

**Figure 6:**
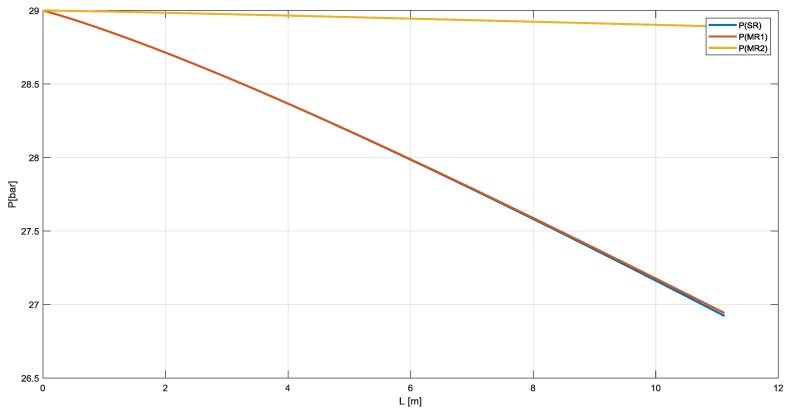
Pressure profile in SR, MR1, and MR2.

In Figure 6, instead, the pressure profile decreases from 29 bar at the inlet of the reactor to 27 bar at the outlet for SR and MR1. Pressure drops are lower for MR2 in which the catalyst is in the annulus section. Furthermore, in this configuration membrane works under compression avoiding the detachment of membrane support. 

**Figure 7:**
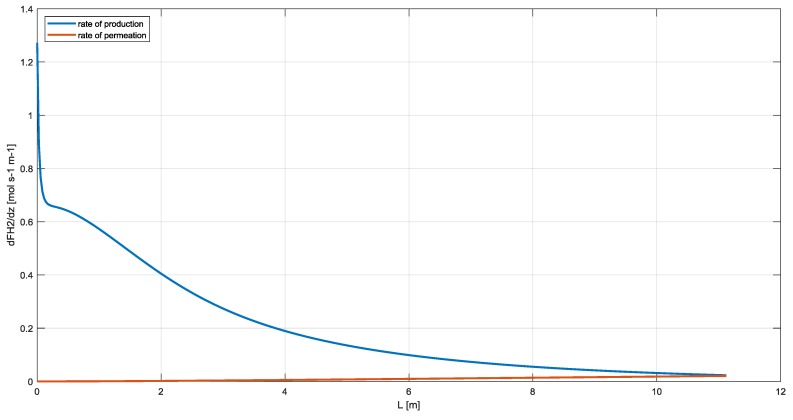
Comparison between the rate of production and rate of permeation of hydrogen in a commercial steam reformer.

However, as shown in Figure 7, the rate of production of hydrogen is about ten times the rate of permeation and, only at the end of the reactor, the two velocities are equal. This justify the use of one-dimensional model also for MR configuration and the necessity to put the membrane outside the reaction section such as RMM scheme. Moreover, as shown in Barba et al., the MR is mechanically complex, requires greater heat transfer surface [28] and the reactor should work at lower temperature of about 850 K to allow membrane thermal stability [43,44]. 

### 3.2. RMM Configuration

In this paragraph the same simulation has been performed with RMM. In this way, the reformer worked as a commercial one and the membrane, out of the reaction section, has been simulated at lower temperature of about 750 K. The syngas at the outlet of the steam reformer SR1 is routed to the membrane M1 at about 27 bar. The separator has been considered as an isobar material exchanger. Hence, the retentate enters the steam reformer SR2 at the same pressure. The removal of a product in the feed allows to shift the equilibrium of the reaction and to convert the residual methane. The hydrogen produced at 25 bar in SR2 is further recovered in membrane M2. For both the membranes the sweeping gas is neglected and the permeate side works at about 1.3 bar. 

**Figure 8:**
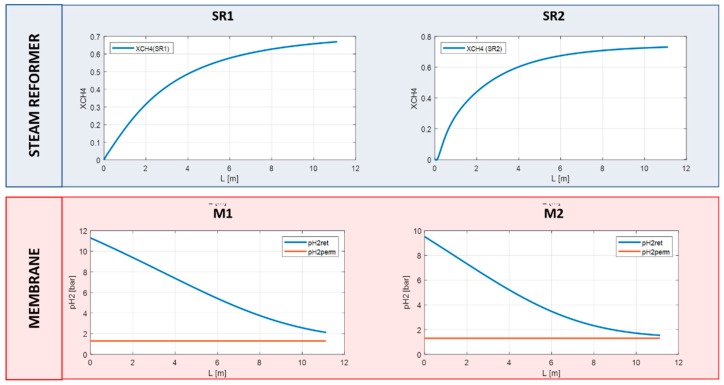
Methane conversion and hydrogen partial pressure in RMM scheme.

**Figure 9:**
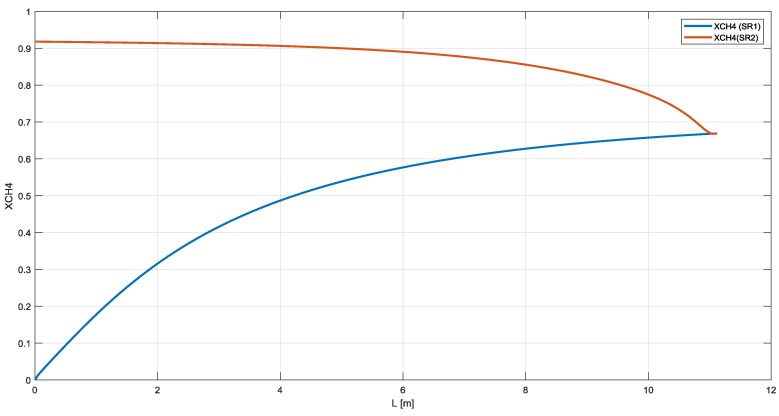
Overall methane conversion in RMM.

As shown in Figure 8, the methane conversion reached 68% in the SR1 and a further 75% in the SR2 with an overall methane conversion of about 92% (Figure 9). The hydrogen recovery factor for membranes M1 and M2 is about 88% and 84%, respectively. 

### 3.3. Feed and Gas Composition for SR, MR, and RMM

**Figure 10:**
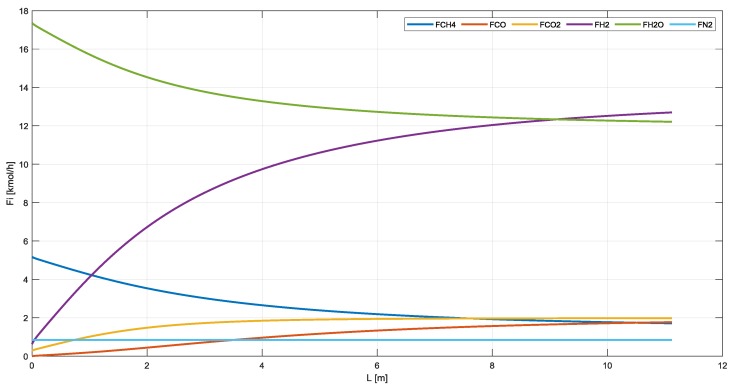
Gas flowrate in SR.

**Figure 11:**
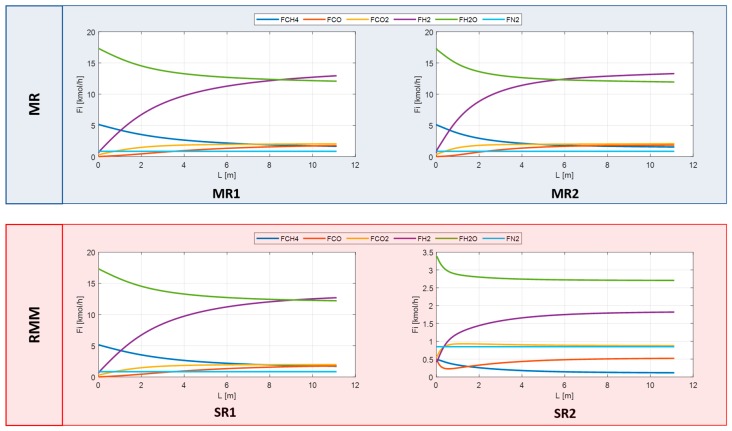
Gas flowrate in MRs and RMM.

The feed used for the simulations is listed in Table 3 and Table 4, where the gas composition at the outlet of the reactors and membranes for the three configurations are summarized. In Figure 10 and Figure 11, instead, the gas flowrates in SR, MR, and RMM are shown.

### 3.4. Sensitivity Analysis

**Figure 12:**
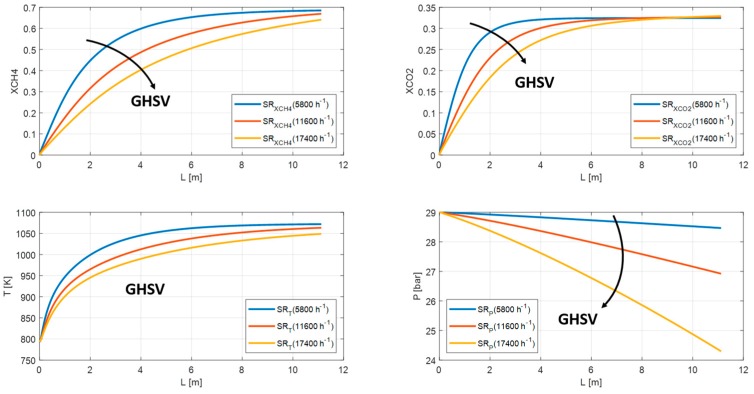
Methane conversion, carbon dioxide yield, temperature, and pressure profile in SR for GHSV = 5800, 11,600, 17,400 h^−1^.

**Figure 13:**
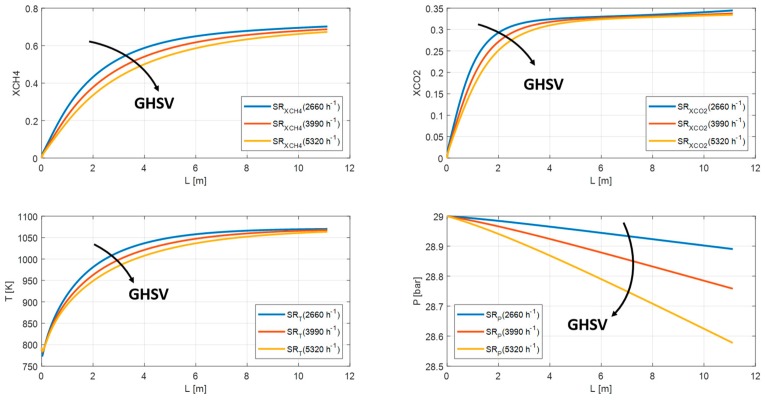
Methane conversion, carbon dioxide yield, temperature, and pressure profile in MR for GHSV = 2660, 3990, 5320 h^−1^.

In this section, the feed composition and geometrical characteristics are fixed and equal to the previous case. The main steam reformer and membrane reactor parameters (GHSV, S/C, and HRF), instead, have been varied. As discussed in the previous paragraph, the SR and MR1 have a similar behavior. Therefore, from this point forward, only the MR2 will be taking into account and will be renamed generically MR. Regarding the increasing GHSV values, both SR and MR show a reduction in methane conversion from 68% to 64% for SR and from 70% to 67% for MR due to unfavorable residence time and temperature profile. The carbon dioxide yield is almost the same; whereas the pressure profile drops owing to increasing volumetric flowrates from 28.5 bar to 24.3 bar for SR and from 28.9 bar to 28.7 bar for MR (Figure 12 and Figure 13).

**Figure 14:**
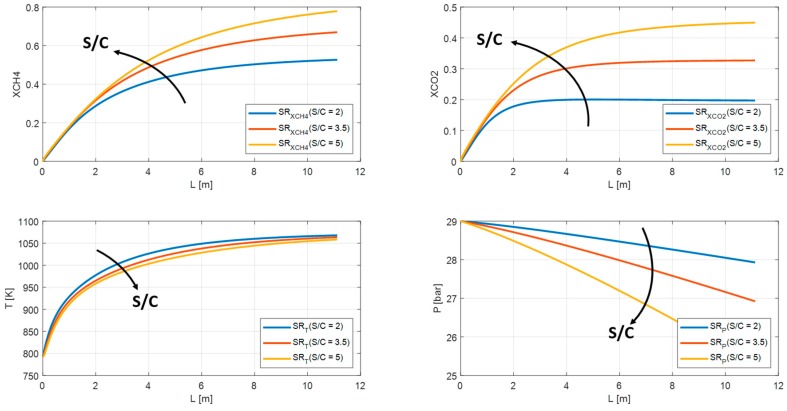
Methane conversion, carbon dioxide yield, temperature, and pressure profile in SR for S/C = 2, S/C = 3.5, S/C = 5.

**Figure 15:**
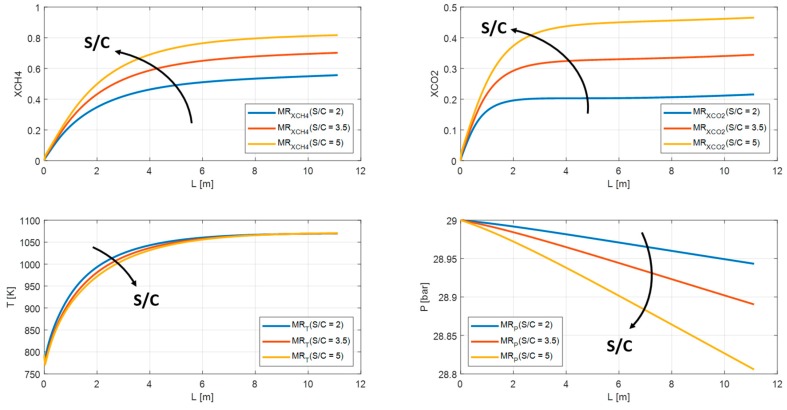
Methane conversion, carbon dioxide yield, temperature, and pressure profile in MR for S/C = 2, S/C = 3.5, S/C = 5.

Both steam reformer and water gas shift reactions are promoted by increasing S/C. As shown in Figure 14 and Figure 15, the methane conversion and carbon dioxide grow from 53% to 78% and from 20% to 45% respectively for SR. Whereas for MR from 56% to 82% for methane conversion and from 22% to 45% for carbon dioxide. The profile temperature can be considered stable, while the pressure profile decreases from 29 bar up to 25 bar for SR and from 28.9 bar to 28.8 bar for MR.

**Figure 16:**
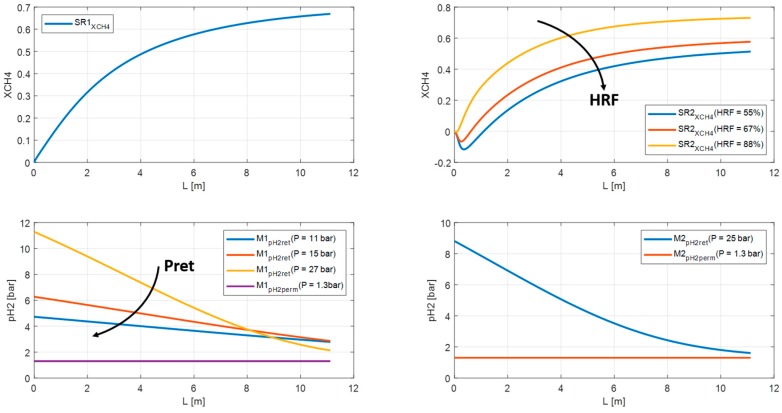
Methane conversion and HRF in RMM at different pressure on retentate side for M1 and M2.

**Figure 17:**
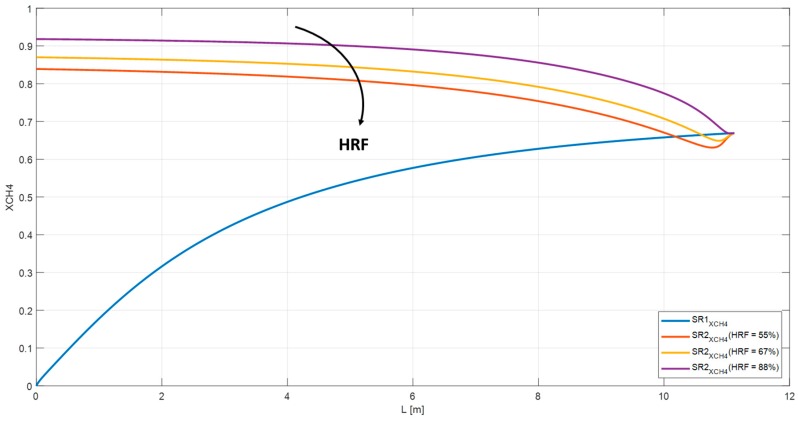
Overall methane conversion in RMM as function of HRF.

The HRF has been examined for the RMM configuration. By decreasing the pressure in the retentate side of the M_1_ membrane from 27 bar to 11 bar, the HRF falls from 88% to 55%. Furthermore, the methane conversion in the SR2 drops from 75% to 51% and the overall conversion in the system from 92% to 84% (Figure 16 and Figure 17). The reduction of methane conversion at the inlet of the SR2 are related to efficiency factors defined in the Xu and Froment equations. Table 5 lists the methane and carbon dioxide conversion for the ‘base case’ analyzed in Section 3. Whereas, the effect of GHSV, S/C, and HRF on the process efficiency is shown in Table 6, Table 7 and Table 8. 

## 4. Conclusions

Hydrogen production has increased four-fold in the last 40 years. Steam reformer of natural gas is still the most common and cheapest way to produce hydrogen; indeed, hydrogen from renewable resources is expensive compared to fossil fuels and except for biomass gasification, the current technologies are at laboratory scale. Moreover, the steam reforming is a key technology for promoting decarbonization of fossil fuels pathways. Pre-combustion carbon-capture strategies based on the reforming of hydrocarbons appear to be the most ready and affordable solution to reduce CO_2_ emissions while waiting for a future energy transition.

In this paper, the commercial steam reformer (SR) has been compared with two emerging technologies: the membrane reactor, with catalyst packed in the tube section (MR1) or in the annuls section (MR2), and the reformer and membrane module (RMM). These architectures allow to increase the production yields, to couple the steam reforming to solar energy harvesting and to realize pre-combustion capture schemes including the separation of CO_2_-rich currents from those rich in hydrogen.

A one-dimensional mathematical model has worked out for mass, heat and momentum balances for the three configurations considering the same operating conditions, void fraction of the bed and geometrical length. The benchmarking between the rate of production and rate of permeation of hydrogen highlighted that only at the end of the reactor the two velocities are equal with an average rate of production ten times greater than the other one. 

The RMM configuration allows to match this phenomenology; indeed, by decoupling the reaction section and permeation one is possible to optimize the two equipment independently. Therefore, the steam reformer has been simulated as commercial one according to Xu and Froment equations; whereas the membrane module has been developed as an isobar and material exchanger working at 750 K. The mass transfer coefficient on retentate side has been estimated according to a previous work where more than 70 tests have been analyzed in the RMM Pilot plant (Chieti, Italy). The results shown an overall methane conversion of 92% with two reactors and membrane modules and a hydrogen recovery factor of 88% and 84% for the two membranes. As shown in the paper, the HRF rises with partial pressure on retentate side of the membrane. This allows to enhance the methane conversion in the second stage of RMM, improving the efficiency of the system. The XCH_4_, indeed, passes from 84% to 92% increasing the pressure on retentate side from 11 bar to 27 bar. In the MR, instead, the methane conversion reaches 68% for MR1 and 70% for MR2. Moreover, for higher GHSV and lower S/C the efficiency of this configuration, in term of methane conversion, decreases up to 56% when S/C = 2. Furthermore, the MR is mechanically complex and requires a pre-reforming section to allow a thermal stability at the inlet of the reactor. Temperature drops, typical of fixed bed reactor, will affect the sealing and the mechanical stability of the membrane. In the RMM, on the contrary, the separator can be designed as a shell and tube configuration instead of an embedded membrane in a catalyst tube. Hence, the RMM could be a starting point for increasing steam reform efficiency and to produce hydrogen, separating carbon dioxide from fossil fuels before combustion.

## Figures and Tables

**Table 1:** Xu and Froment kinetic parameters.

Reactions	Rates of Reactions	Kinetic Parameters
$CH_{4}+H_{2}O⇄CO+3H_{2}$	$r_{1}=\frac{\frac{k_{1}}{p_{H_{2}}^{2.5}}\left(p_{CH_{4}}p_{H_{2}O}-\frac{p_{H_{2}}^{3}p_{CO}}{K_{1}}\right)}{{\left(DEN\right)}^{2}}$	$k_{1}=4.225\cdot 10^{15}e^{-\frac{28879}{T}}$
$CO+H_{2}O⇄CO_{2}+H_{2}$	$r_{2}=\frac{\frac{k_{2}}{p_{H_{2}}}\left(p_{CO}p_{H_{2}O}-\frac{p_{H_{2}}p_{CO_{2}}}{K_{2}}\right)}{{\left(DEN\right)}^{2}}$	$k_{2}=1.955\cdot 10^{6}e^{-\frac{8074.3}{T}}$
$CH_{4}+2H_{2}O⇄CO_{2}+4H_{2}$	$r_{3}=\frac{\frac{k_{3}}{p_{H_{2}}^{3.5}}\left(p_{CH_{4}}p_{H_{2}O}^{2}-\frac{p_{H_{2}}^{4}p_{CO_{2}}}{K_{3}}\right)}{{\left(DEN\right)}^{2}}$	$k_{3}=1.02\cdot 10^{5}e^{-\frac{29336}{T}}$
**Equilibrium Constant**	**Adsorption Constant**	
$K_{1}=1.01325^{2}\cdot e^{-\frac{53717-60.25\cdot T}{1.987\cdot T}}$	$K_{CO}=8.23\cdot 10^{-5}e^{\frac{8497.7}{T}}$	
$K_{2}=e^{-\frac{-8514+7.11\cdot T}{1.987\cdot T}}$	$K_{CH_{4}}=6.65\cdot 10^{-4}e^{\frac{4604.3}{T}}$	
$K_{3}=1.01325^{2}e^{-\frac{45203-52.54\cdot T}{1.987\cdot T}}$	$K_{H_{2}}=6.12\cdot 10^{-9}e^{\frac{9971.13}{T}}$	
-	$K_{H_{2}O}=1.77\cdot 10^{5}e^{-\frac{10666.35}{T}}$	

where $DEN=1+K_{CO}p_{CO}+K_{H_{2}}p_{H_{2}}+K_{CH_{4}}p_{CH_{4}}+\frac{K_{H_{2}O}p_{H_{2}O}}{p_{H_{2}}}$.

**Table 2:** Data [27,38,39].

Parameters	SR	MR	RMM (Reformer and Membrane Module)
*P^R^*, bar	29	29	29
*P^P^*, bar	-	1.3	1.3
*T*, K	793.15	793.15	793.15
*Tw*, K	1073	1073	1073
*Lr*, m	11.12	11.12	11.12
*L^M^*, m	11.12	11.12	11.12
*ODs*, m	-	0.28	0.28
*IDs*, m	-	0.25	0.25
*IDt*, m	0.1016	0.1016	0.1016
*ODt*, m	0.1322	0.1322	0.1322
*dc*, m	0.02	0.02	0.02
*ρ_c_*, kg_c_·m_r_^−3^	2355.2	2355.2	2355.2
*kc*, J·s^−1^·m^−1^·K^−1^	3.8	3.8	3.8
*kt*, J·s^−1^·m^−1^·K^−1^	43	43	43
$E_{a},$ kJ·mol^−1^	-	20.2	20.2
$P_{H_{2}}^{0}$, kmol·h^−1^·m^−1^·bar^−0.5^	-	1.69 × 10^−4^	1.69 × 10^−4^
$\bar{K_{H_{2}}}$, kmol·h^−1^·m^−2^·bar^0.5^	-	1.92	1.92

**Table 3:** Gas composition for SR and MRs.

Component	FEED		SR		MR1		MR2	
	In		Out		Out		Out	
	kmol/h	% mol	kmol/h	% mol	kmol/h	% mol	kmol/h	% mol
CH_4_	5.17	21.28%	1.71	5.48%	1.65	5.26%	1.54	4.88%
CO	0.00	0.00%	1.77	5.67%	2.05	6.53%	2.07	6.56%
CO_2_	0.29	1.19%	1.98	6.34%	1.77	5.63%	1.85	5.86%
H_2_	0.63	2.60%	12.70	40.68%	12.95	41.33%	13.30	42.14%
H_2_O	17.36	71.45%	12.21	39.12%	12.08	38.55%	11.95	37.87%
N_2_	0.85	3.49%	0.85	2.72%	0.85	2.71%	0.85	2.69%

**Table 4:** Gas composition for RMM.

Component	FEED		SR1		M1		SR2		M2	
	In		Out		Out		Out		Out	
	kmol/h	% mol	kmol/h	% mol	kmol/h	% mol	kmol/h	% mol	kmol/h	% mol
CH_4_	5.17	21.28%	1.71	5.48%	1.71	8.54%	0.42	1.87%	0.42	2.47%
CO	0.00	0.00%	1.77	5.67%	1.77	8.84%	1.88	8.31%	1.88	10.97%
CO_2_	0.29	1.19%	1.98	6.34%	1.98	9.88%	3.16	13.97%	3.16	18.44%
H_2_	0.63	2.60%	12.70	40.68%	1.51	7.54%	6.55	28.98%	1.07	6.27%
H_2_O	17.36	71.45%	12.21	39.12%	12.21	60.97%	9.75	43.11%	9.75	56.90%
N_2_	0.85	3.49%	0.85	2.72%	0.85	4.23%	0.85	3.75%	0.85	4.95%

**Table 5:** Methane conversion, carbon dioxide yield, and hydrogen recovery factor at fixed GHSV (11,600 h^−1^ SR and 2660 h^−1^ MR), S/C (3.5) and membrane area (4.6 m^2^).

KEY PARAMETERS	SR	MR	RMM
XCH_4_	67%	70%	92%
XCO_2_	33%	35%	55%
HRF	-	-	88%

**Table 6:** Effect of GHSV in SR and MR.

**SR.**	**GHSV (5800 h^−1^)**	**GHSV (11,600 h^−1^)**	**GHSV (17,400 h^−1^)**
XCH_4_	68%	67%	64%
XCO_2_	32%	33%	33%
**MR**	**GHSV (2660 h^−1^)**	**GHSV (3900 h^−1^)**	**GHSV (5320 h^−1^)**
XCH_4_	70%	69%	67%
XCO_2_	34%	34%	33%

**Table 7:** Effect of S/C in SR and MR.

**SR**	**S/C = 2**	**S/C = 3.5**	**S/C = 5**
XCH_4_	53%	67%	78%
XCO_2_	20%	33%	45%
**MR**	**S/C = 2**	**S/C = 3.5**	**S/C = 5**
XCH_4_	56%	70%	82%
XCO_2_	22%	34%	45%

**Table 8:** Effect of pressure on retentate side in RMM.

RMM	Pret (11 bar)	Pret (15 bar)	Pret (27 bar)
XCH_4_	84%	87%	92%
XCO_2_	46%	49%	55%
HRF	55%	67%	88%

## Nomenclature

$c_{p}$	specific heat (J·kg^−1^·K^−1^);
$d_{c}$	catalytic pellets diameter (m);
$d_{i}$	internal shell/tube diameter for MR and SR respectively (m);
$d_{e}$	outside shell/tube diameter for MR and SR respectively (m);
$D_{0,H}$	diffusion pre-exponential factor (m^2^·s^−1^);
$E_{a}$	activation energy for hydrogen permeation through metallic membranes (J·mol^−1^);
$E_{D}$	activation energy for the diffusion of hydrogen atoms (J·mol^−1^);
${\left(\frac{dF_{H_{2}}}{dz}\right)}_{prod.}$	rate of production of hydrogen (mol·m^−1^·s^−1^);
${\left(\frac{dF_{H_{2}}}{dz}\right)}_{perm.}$	rate of permeation of hydrogen (mol·m^−1^·s^−1^);
*f*	friction factor;
$F_{G}$	mass transfer coefficient (kmol·h^−1^·m^−2^);
$F_{OG}$	overall mass transfer coefficient (kmol·h^−1^·m^−2^);
*GHSV*	gas hourly space velocity (h^−1^);
$h_{i}$	convective heat transfer coefficient in packed bed (J· m^−1^·s^−1^·k^−1^);
$ID_{s}$	internal shell diameter (m);
$ID_{t}$	internal tube diameter (m);
$J_{H}{}_{2}$	hydrogen molar flux (mol·m^−2^·s)
$k_{T}$	thermal conductivity of tube (J·s^−1^·m^−1^·K^−1^);
$k_{c}$	thermal conductivity of catalyst (J·s^−1^·m^−1^·K^−1^);
$k_{1}$	steam reformer reaction rate constant;
$k_{2}$	water gas shift reaction rate constant;
$k_{3}$	overall steam reformer reaction rate constant;
$K_{1}$	steam reformer equilibrium constant (bar^2^);
$K_{2}$	water gas shift equilibrium constant;
$K_{3}$	overall steam reformer equilibrium constant (bar^2^);
$K_{CO}$	carbon monoxide adsorption equilibrium constant;
$K_{CH_{4}}$	methane adsorption equilibrium constant;
$K_{H_{2}}$	hydrogen adsorption equilibrium constant;
$K_{H_{2}O}$	steam adsorption equilibrium constant;
$\bar{K_{H_{2}}}$	average hydrogen permeance (kmol· h^−1^·m^−2^·bar^−0.5^);
L	reactor, membrane geometrical length (m);
$OD_{s}$	outside shell diameter (m);
$OD_{t}$	outside tube diameter (m);
P	operating pressure (bar);
$p_{CO}$	carbon monoxide partial pressure (bar);
$p_{CH_{4}}$	methane partial pressure (bar);
$p_{CO_{2}}$	carbon dioxide partial pressure (bar);
$p_{H_{2}O}$	steam partial pressure (bar);
$p_{H_{2}}$	hydrogen partial pressure (bar);
$P_{H_{2}}^{0}$	permeability pre-exponential factor (kmol·h^−1^·m^−1^·bar^−0.5^);
$P_{H_{2}}$	hydrogen permeability (kmol·h^−1^·m^−1^·bar^−0.5^);
$r_{1}$	steam reformer reaction rate (mol·kg_c_^−1^·s^−1^);
$r_{2}$	water gas shift reaction rate (mol·kg_c_^−1^·s^−1^);
$r_{3}$	overall steam reformer reaction rate (mol·kg_c_^−1^·s^−1^);
$r_{i}$	rate of disappearance of i-th reactions (kmol_i-th_·kg_c_^−1^·h^−1^);
$r_{CH_{4}}$	rate of disappearance of methane (kmol_CH4_·kg_c_^−1^·h^−1^);
$r_{CO_{2}}$	rate of production of carbon dioxide (kmol_CH4_·kg_c_^−1^·h^−1^);
$r_{H_{2}}$	rate of production of hydrogen (kmol_CH4_·kg_c_^−1^·h^−1^);
Re	Reynolds number;
*S/C*	steam to carbon ratio;
T	operating temperature (K);
$T_{w}$	tube wall temperature (K);
u	superficial velocity of gas mixture (m^3^·m^−2^·s^−1^);
U	overall mass transfer coefficient (J·m^−2^·K^−1^·s^−1^);
$X_{CH_{4}}$	methane conversion;
$X_{CO_{2}}$	carbon dioxide yield;
**Apices and Subscripts**	
c	relative to the catalyst;
*LI*	relative to the left interface in the film theory;
*M*	relative to the membrane;
*ML*	logarithm mean;
*P*	relative to permeate side;
R	relative to the retentate side;
RI	relative to the right interface in the film theory;
*r*	relative to reactor;
s	relative to the shell-side;
*t*	relative to tube-side;
$z_{i}$	axial coordinates of mass/heat/momentum balances;
**Greek Letters**	
ε	void fraction of the bed;
δ	membrane thickness, (m);
$\Delta H_{i}$	molar enthalpy of *i*-th reactions (J·mol^−1^);
$\Delta H_{R}^{0}$	standard enthalpy of the surface dissociation reaction (J·mol^−1^);
$\Delta S_{R}^{0}$	entropy change of the dissociation reaction (J·mol^−1^·K^−1^);
$\eta_{i}$	efficiency factor of *i*-th reactions;
$\eta_{CH_{4}}$	methane efficiency factor;
$\eta_{CO_{2}}$	carbon dioxide efficiency factor;
$\rho_{g}$	density of gas mixture (kg·m^−3^);
$\rho_{c}$	density of catalytic bed (kg_c_·m^−3^);
*Ω*	cross section of the reactor (m^3^)
